# Experimental silicosis does not aggravate collagen-induced arthritis in mice

**DOI:** 10.1186/s12952-017-0071-6

**Published:** 2017-03-13

**Authors:** Robby Engelmann, Brigitte Müller-Hilke

**Affiliations:** Institute of Immunology & Core Facility for Cell Sorting & Cell Analysis, Rostock University Medical Center, Schillingallee 70, 18057 Rostock, Germany

**Keywords:** Mucosal immunity, Collagen-induced arthritis, Silicosis

## Abstract

**Objectives:**

To investigate the effect of chronic lung inflammation on the incidence and severity of collagen-induced arthritis in mice.

**Methods:**

Chronic lung inflammation in the form of silicosis was induced via intranasal application of silica particles. Immunization with collagen Type II commenced one week later and mice were sacrificed six weeks after booster immunization. Thereafter, silicosis was confirmed via flow cytometry and arthritis was evaluated performing knee and paw histology.

**Results:**

Pronounced lung inflammation in the silica-treated compared to PBS-treated control mice was demonstrated by significantly elevated broncho-alveolar lavage (BAL) cell count, attributable to increased numbers of macrophages and granulocytes. Inflammation in the lungs was not associated with elevated PAD2 and PAD4 expression, yet silica treated animals had significantly higher aCCP serum titers. However, lung inflammation did not lead to an increase in the incidence of arthritis, nor did it exacerbate the macroscopic or histologic joint scores.

**Conclusions:**

Chronic lung inflammation resulting from silicosis does not aggravate collagen-induced arthritis in mice.

**Electronic supplementary material:**

The online version of this article (doi:10.1186/s12952-017-0071-6) contains supplementary material, which is available to authorized users.

## Background

Rheumatoid arthritis (RA) is a systemic autoimmune disease which manifests predominantly as a chronic polyarthritis. The autoantibodies against citrullinated antigens (ACPA) have become indispensable for RA diagnosis and define a distinct subset of RA for which a variety of genetic and environmental risk factors have been described. While the strongest genetic risk is conferred by genes encoding the major histocompatibility complex (HLA), environmental risk factors include smoking, exposure to silica particles and air pollution as well as periodontitis and the recently discussed gastrointestinal microbiota [1, 2].

Among these, smoking is the most extensively studied risk factor for RA. It predisposes for ACPA-positive RA in subjects carrying the shared HLA epitope [3]. Not only the expression of the citrullinating enzyme peptidylarginine deiminase (PAD) 2 but also citrullinated proteins are elevated in the lungs of smokers compared to non-smokers [4]. These findings suggested that smoking causes an increase in citrullination which eventually leads to the breach in tolerance. However, a recent report demonstrated that it is not the cigarette smoke per se that increases citrullination in the lungs. In fact, it appears that it is inflammation associated with smoking that is responsible for elevated levels of citrullinated lung proteins as the latter were also demonstrated for patients with chronic obstructive pulmonary disease (COPD) [5]. We therefore set out to investigate the effect of silicosis - a model for chronic lung inflammation - on collagen-induced arthritis, a murine model for RA.

## Methods

### Mice

Female DBA/1J (Charles River, Wilmington, MA, USA) and male B10.q (Jackson) mice were crossed. Parental and F1 mice were maintained in a specific pathogen free unit on a 12 h light/12 h dark cycle with 30 min twilight period. The ambient temperature was 21 ± 2°C, the humidity was 60 ± 10% and the room air change is 20-fold. Mice were housed using a stocking density of 3–5 mice per cage. Mice were given water and ssniff R/M-H diet (ssniff Spezialdiäten GmbH, Soest, Germany) ad libitum.

### Silicosis

A solution of 40 mg silica particles [particle size range: 0.5–10 μm, 80% are 1–5 μm] (Sigma Aldrich, St. Louis, MO, USA) per ml PBS was prepared and sonicated for 10 min prior to use. Male F1 mice aged 6–12 weeks were anesthetized by i.p. injection of 75 mg/kg ketamine (bela-pharm GmbH & Co. KG, Vechta, Germany) and 6 mg/kg xylazine (Bayer AG, Leverkusen, Germany). Two droplets of 15μl of silica solution each were subsequently applied onto the noses of the anesthetized animals. Controls were given the same amount of PBS alone. The suspension was inhaled involuntarily and mice developed pulmonary inflammation as described previously [6].

### Collagen-induced arthritis

Collagen-induced arthritis was induced one week after intranasal treatment with silica particles or PBS. Bovine collagen type II (MD Bioscience, St. Paul, MN, USA) was dissolved at a concentration of 2 mg/ml in sterile 0.1M acetic acid by stiring overnight at 4°C. Complete Freunds’ adjuvant (CFA) was prepared by thoroughly mixing 25 ml incomplete Freunds’ adjuvant (IFA) (Difco, Detroit, MI, USA) with 100 mg *M. tuberculosis* H37 RA (Difco, Detroit, MI, USA). For the primary immunization 400 μl CFA was mixed with 400 μl collagen solution and 100 μl/mouse were injected intra-dermally at the tail base. Booster immunization was performed 3 weeks after primary immunization using IFA instead of CFA. Mice were scored weekly, following an extended scoring protocol whereby each paw was scored for macroscopic signs of arthritis. Each affected distal joint of the toe/knuckle scored one point and affected midpaws/ankles scored five points. Thus, each paw can reach a maximum score of 15 and each mouse a maximum score of 60.

### Evaluation of silicosis

Six weeks after booster immunization, animals were anesthetized and broncho-alveolar lavage cells were isolated by flushing the lungs 3 times with 800 μl PBS pH 7.4 containing 0.1 mM EDTA. Collected cells were counted in a hemocytometer and subsequently centrifuged at 300×g for 10 min. Cell pellets were then resuspended in 100 μl ice-cold PBS pH 7.4, 0.5% bovine serum albumin, 0.1% sodium azide and therein stained for CD11c: FITC (clone HL3, BD, Franklin Lakes, NJ, USA), CD45:PE (clone 30-F11, Biolegend, San Diego, CA, USA), GR-1: PECy7 (clone RB6-8C5, Biolegend, San Diego, CA, USA) and IA^q^: Alexa647 (clone KH116, Biolegend, San Diego, CA, USA). Cells were analyzed in a BD FACS Calibur (BD, Franklin Lakes, NJ, USA).

### Histology

Paws and knees were excised and fixed in 4% paraformaldehyde for 5 days. Paraformaldehyde was removed under floating tap water for 30 min and tissues were transferred into USEDECALC (Medite GmbH, Burgdorf, Germany) for decalcification for 5 days (knees) or 2 weeks (paws). Tissue samples were paraffin-embedded and 5 μm thin-sections were made. Sections were deparaffinized and rehydrated prior to staining with haematoxylin/eosin. Stained sections were scored on an Axioplan 2 microscope (Carl Zeiss AG, Oberkochen, Germany) using a previously published scoring system [7]. In brief, knee joints we scored for inflammation (evaluating the degree of infiltration yielding a score between 0 and 3), cartilage destruction (normal via empty lacunae up to complete loss of articular cartilage, again yielding a score between 0 and 3) and bone loss (yielding scores between 0 and 5). The total of all three parameters will result in a maximum score of 11. The paws were graded differently and for the parameters pannus severity, cellular infiltration, cartilage destruction and bone loss, each yielding a score between 0 and 4. Each paw could thus reach a maximum score of 16 and all four paws per mouse were averaged.

### Serum antibodies

ACPA IgG levels were measured by combining CCP-(Euroimmun, Lübeck, Germany; CCP2) and MCV-(Orgentec, Mainz, Germany) coated ELISA plates with an anti-mouse IgG antibody coupled to horse radish peroxidase (STAR13B; Bio-Rad Laboratories, Hercules, CA, USA). The sera were applied at a dilution of 1:50 for 1h at RT. Thereafter, the plates were incubated with the detection antibody at a dilution of 1:1000 for 1h. Finally, color reaction was performed using TMB substrate (Biolegend, Fell, Germany) and the optical densities were determined by an automated plate reader (Millenia Kinetic Analyser, DPC, USA). Antibody serum levels against collagen type II were analyzed by coating Nunc MediSorp ELISA plates (Thermo Fisher Scientific, Waltham, MA, USA) with bovine collagen type II (MD Bioscience, St. Paul, MN, USA) at 20μg/ml in carbonate/bicarbonate buffer overnight at 4°C. Sera were applied at a dilution of 1:12,000 for 1.5h at RT and bound antibodies were detected as described for ACPA detection.

### Statistics

For normal distributed data means and SEM are shown. Otherwise medians and quartiles are used. Means were compared by Student’s *t*-test and medians were compared by Mann Whitney *U*-test. *P*-values for the time course of CIA were calculated either by Fisher Test (incidence) or by Mann–Whitney *U*-Test (macroscopic score) for each time point separately. Statistics were performed using R (v3.2.2).

## Results

**Fig. 1:**
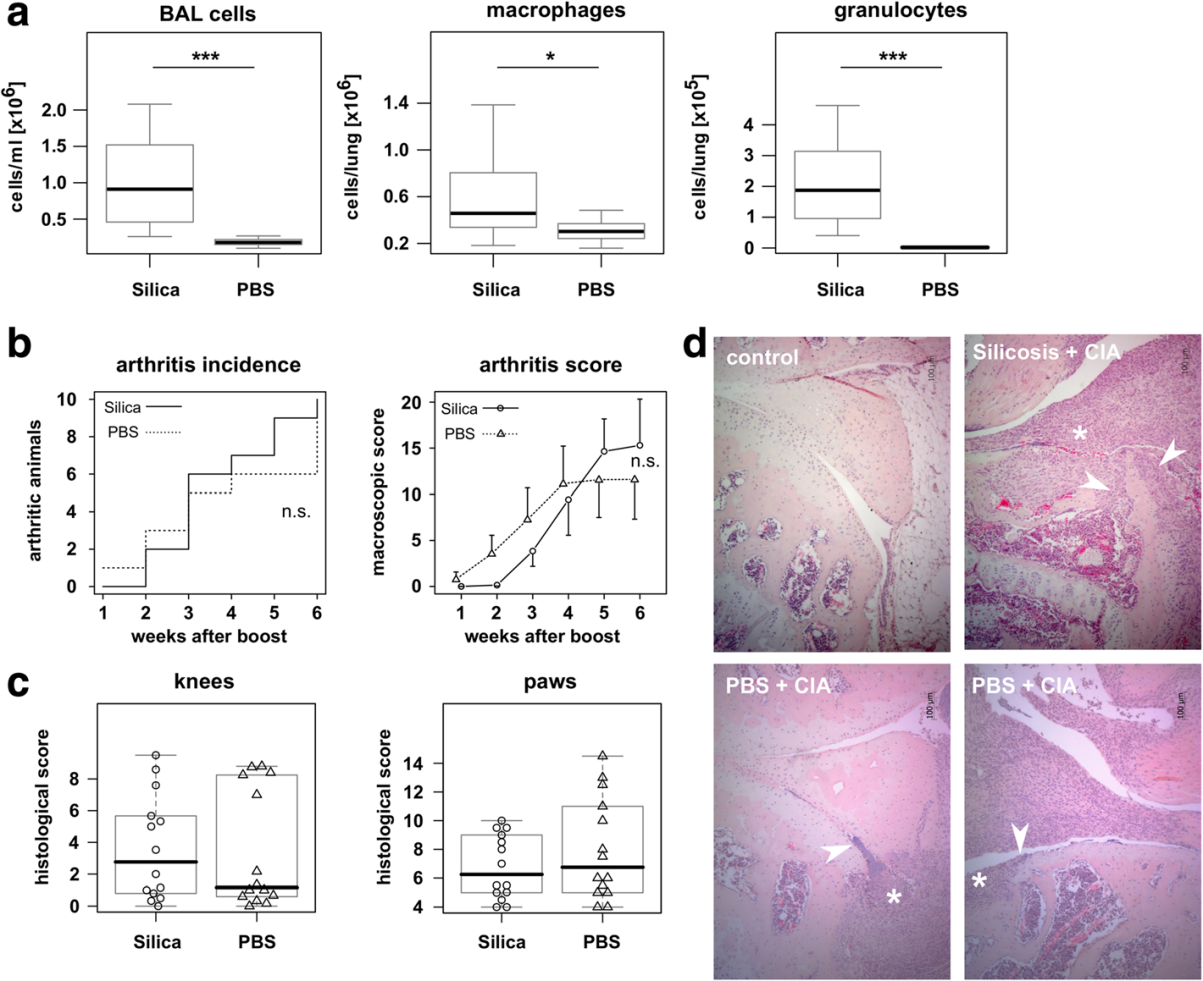
Silicosis does not aggravate collagen-induced arthritis. **a** BAL cells from mice six weeks after booster immunization with collagen type II were isolated and phenotyped by flow cytometry for macrophages (CD45^+^IAq^+^GR-1^−^CD11c^+^) and granulocytes (CD45^+^IAq^−^GR-1^+^CD11c^−^). Boxes show medians and quartiles, p-values were calculated by Mann–Whitney *U*-test. n = 14. **b** Neither the arthritis incidence (left panel) nor the macroscopic arthritis score (right panel) differ between silica-treated and control mice. *p*-values were calculated by Fisher Test (incidence) and Mann–Whitney *U*-Test (macroscopic score) for each time point. **c** Histological scores for knee joints and paws do not differ between silica-treated and control mice. Boxes show median and quartiles, each dot represents one mouse. P-values were calculated by Mann–Whitney *U*-Test. **d** Examples for the H&E staining of thin-sections from femorotibial joints in control mice without immunization with collagen type II (upper left panel), silica-treated mice immunized with collagen type II (upper right panel) and PBS-treated mice immunized with collagen type II (lower panels) showing a high (lower right panel) or a low (lower left panel) histological score are shown. Asterisks indicate inflammatory infiltrates. Arrowheads point at sites of cartilage or bone destruction

Intranasal application of silica particles in mice led to a longstanding inflammation. The broncho-alveolar lavage (BAL) of silica treated and control mice was analyzed ten weeks after the induction of silicosis and indeed, there was a significant increase in the total numbers of BAL cells (*p* < 0.001), in particular macrophages (*p* = 0.03) and granulocytes (*p* < 0.001) after silica-treatment (Fig. 1a). Despite the longstanding inflammation, we could not detect a difference in the expression of PAD2 and PAD4 between silica treated and PBS treated mice (Additional file 1: Figure S1).

In contrast, silicosis had no impact on the incidence and severity of collagen induced arthritis. Primary immunization with collagen commenced one week after silica treatment and mice were then followed up for the rest of the observation period. At the final time point, six weeks after booster immunization, there were no differences between the silicosis and the control groups with respect to arthritis incidence and severity, the latter being scored macroscopically as well as histologically for knees and paws (Fig. 1b, c, histology is exemplified in Fig. 1d). Likewise, there was no correlation between the numbers of total BAL cells, BAL macrophages or BAL granulocytes and the macroscopic arthritis score (Additional file 1: Figure S1, data not shown) nor did we find significant differences in anti-collagen type II and anti-MCV IgG serum level comparing silica treated and PBS treated mice. However, we did find significant difference between silica treated and PBS treated mice for anti-CCP IgG serum level (*p* = 0.02; Additional file 1: Figure S1) and we also found a significant correlation between anti-collagen type II IgG and the macroscopic arthritis score (R = 0.65, *p* < 0.001). We therefore conclude that silica induced chronic lung inflammation in the mouse does not aggravate collagen induced arthritis.

## Discussion

Over the last years, studies accumulated on the genetic and environmental risk factors for RA and on factors driving the disease. Nevertheless, it is still unclear where and by which mechanisms the first break of tolerance occurs [8]. The mucosal surfaces and mucosa associated lymphoid tissues (MALT) are likely to play critical roles as the most prominent risk factors for RA - smoking, periodontitis, gastrointestinal microbiota and silicosis - affect mucosal immunity. We therefore made use of a murine silicosis model to induce a chronic lung inflammation. Despite this chronic inflammatory reaction in the lungs we did not see an exacerbation of the CIA. However, this may be due to the timing between silicosis induction and CIA induction. We decided for a one week interval between silicosis induction and primary immunization with collagen in order to ensure that a full blown inflammation has been established. At five weeks after silicosis treatment – which corresponds to one week after the booster immunization – arthritis starts to be macroscopically visible and silicosis is in its chronic phase. Indeed, it has previously been shown that inflammatory markers increased already one day after the treatment with silica particles, peaking at 5 days and lasting up to 60 days with fibrosis evident at 15 days after silicosis induction [9–11]. Thus, our protocol covers all phases of silicosis.

It is unknown which factors influence the interaction between silicosis and arthritis. However, it has been described that silica treatment induces apoptosis in macrophages, which in turn increase citrullination due to elevated intracellular calcium concentrations [4, 12]. An increase in citrullination may aggravate arthritis as described for the administration of citrullinated collagen [13]. On the other side, it is known that silica treatment can recruit CD4 + Foxp3+ regulatory T cells to the lungs which may counteract immune activation [14]. Although we did not find increased PAD2/4 expression levels in the lungs after silica treatment an increase in intracellular calcium levels would still lead to citrullination by PAD enzymes which is in line with slightly elevated anti-CCP levels in our silica treated mice. However, these differences did not influence the severity of arthritis.

For the induction of CIA male sex and the major histocompatibility allel A^q^ are crucial factors, which are all met in our model [15–17]. Likewise, complex interactions between genetics and the immune system hold true for the development of silicosis [18, 19]. Thus, by focusing on a mouse strain that is highly susceptible for CIA we may have missed factors that are essential for an interaction between silicosis and arthritis. Furthermore, we cannot rule out that a specific activation of pattern-recognition receptors contributes to CIA development yet is lacking from silicosis under specific pathogen free conditions.

## Conclusions

In summary our results suggest that factors other than chronic inflammation in mucosal environments are necessary to compromise immune tolerance in the murine model of CIA. Such factors may well include genetic polymorphisms as previously shown for the gene-environment interaction between the HLA shared epitope and smoking in human ACPA-positive RA [3].

## Additional files

Additional file 1: Figure 2. Additional information. (A) Before we did the combination of silicosis and CIA we performed a time course experiment with silicosis induction only. Eight weeks after silica or PBS treatment we sacrificed the mice and analyzed the mRNA expression of PAD2 and PAD4 in lung tissue. We did not find significant differences between the treatment and control groups nor during the time course of silicosis. (B) H&E stainings of the lung exemplify healthy tissue (upper panel) in PBS treated mice and lung tissue with ongoing inflammation (asterisk, lower panel) in silica treated mice. (C) Serum IgG antibody levels against mutated citrullinated vimentin (MCV), cyclic-citrullinated peptides (CCP) and collagen type II (Col2) show significantly higher levels of anti-CCP IgG among silica treated mice as compared to PBS treated mice (same animals as in Fig. 1). There is no difference between both groups for anti-MCV and anti-Col2 IgG. (D) No correlation exists between the number of BAL granulocytes and the macroscopic arthritis score. Spearman correlation (*R* = 0.22, *p* = 0.3). However, anti-Col2 IgG levels are significantly correlated with the macroscopic arthritis score. Spearman correlation (*R* = 0.65, *p* < 0.001) (same animals as in Fig. 1). (TIFF 2253 kb)
Additional file 2: Original data. (CSV 5 kb)
