# Supplementary figures and images for: Experimental silicosis does not aggravate collagen-induced arthritis in mice
Source: J Negat Results Biomed. 2017 Mar 13;16:5. doi: 10.1186/s12952-017-0071-6 (PMC5346855; doi:10.1186/s12952-017-0071-6)

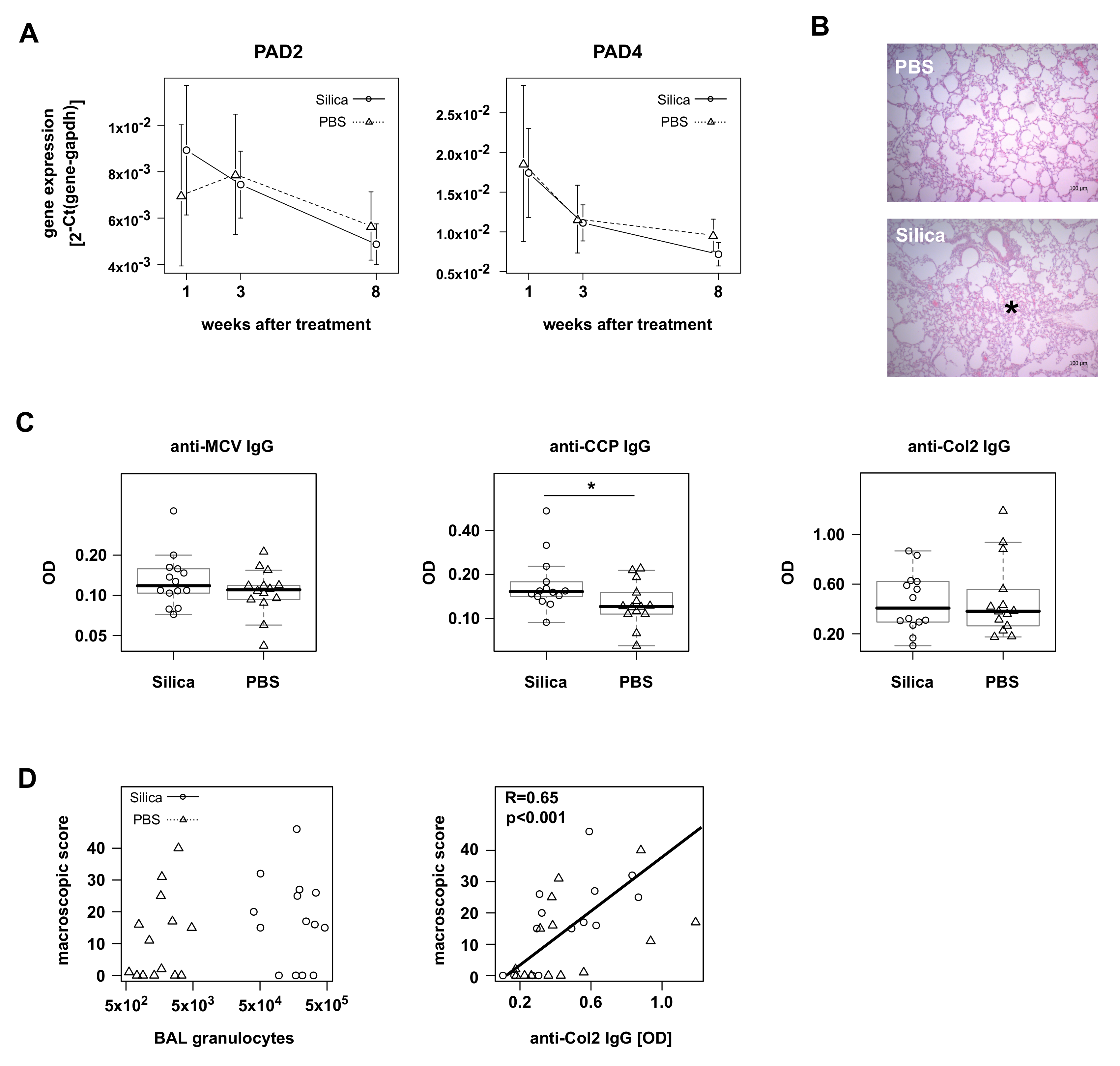

Supplement: Additional file 1: Figure 2. — Additional information. (A) Before we did the combination of silicosis and CIA we performed a time course experiment with silicosis induction only. Eight weeks after silica or PBS treatment we sacrificed the mice and analyzed the mRNA expression of PAD2 and PAD4 in lung tissue. We did not find significant differences between the treatment and control groups nor during the time course of silicosis. (B) H&E stainings of the lung exemplify healthy tissue (upper panel) in PBS treated mice and lung tissue with ongoing inflammation (asterisk, lower panel) in silica treated mice. (C) Serum IgG antibody levels against mutated citrullinated vimentin (MCV), cyclic-citrullinated peptides (CCP) and collagen type II (Col2) show significantly higher levels of anti-CCP IgG among silica treated mice as compared to PBS treated mice (same animals as in Fig. 1). There is no difference between both groups for anti-MCV and anti-Col2 IgG. (D) No correlation exists between the number of BAL granulocytes and the macroscopic arthritis score. Spearman correlation (R = 0.22, p = 0.3). However, anti-Col2 IgG levels are significantly correlated with the macroscopic arthritis score. Spearman correlation (R = 0.65, p < 0.001) (same animals as in Fig. 1). (TIFF 2253 kb) [file 12952_2017_71_MOESM1_ESM.tiff]
